# *BAP1* and *PTEN* mutations shape the immunological landscape of clear cell renal cell carcinoma and reveal the intertumoral heterogeneity of T cell suppression: a proof-of-concept study

**DOI:** 10.1007/s00262-022-03346-7

**Published:** 2022-12-23

**Authors:** Jana Friedhoff, Felix Schneider, Christina Jurcic, Volker Endris, Martina Kirchner, Angela Sun, Iulia Bolnavu, Laura Pohl, Miriam Teroerde, Maximilian Kippenberger, Constantin Schwab, Adam Kaczorowski, Stefanie Zschäbitz, Dirk Jäger, Markus Hohenfellner, Albrecht Stenzinger, Anette Duensing, Stefan Duensing

**Affiliations:** 1 Department of Urology, Molecular Urooncology, University Hospital Heidelberg, Im Neuenheimer Feld 517, 69120 Heidelberg, Germany; 2 Institute of Pathology, University Hospital Heidelberg, Im Neuenheimer Feld 224, 69120 Heidelberg, Germany; 3 Cancer Therapeutics Program, UPMC Hillman Cancer Center, 5117 Centre Avenue, Pittsburgh, PA 15213 USA; 4 Department of Medical Oncology, National Center for Tumor Diseases (NCT), University Hospital Heidelberg, Im Neuenheimer Feld 460, 69120 Heidelberg, Germany; 5 Department of Urology, National Center for Tumor Diseases (NCT), University Hospital Heidelberg, Im Neuenheimer Feld 420, 69120 Heidelberg, Germany; 6 Department of Pathology, School of Medicine, University of Pittsburgh, 200 Lothrop Street, Pittsburgh, PA 15213 USA; 7 Department of Urology, Precision Oncology of Urological Malignancies, University Hospital Heidelberg, Im Neuenheimer Feld 517, 69120 Heidelberg, Germany

**Keywords:** Renal cell carcinoma, *BAP1* mutation, *PTEN* mutation, Tumor-infiltrating lymphocytes (TILs), CD8, FOXP3, CD163

## Abstract

Clear cell renal cell carcinoma (ccRCC) is an immunologically vulnerable tumor entity, and immune checkpoint inhibitors are now widely used to treat patients with advanced disease. Whether and to what extent immune responses in ccRCC are shaped by genetic alterations, however, is only beginning to emerge. In this proof-of-concept study, we performed a detailed correlative analysis of the mutational and immunological landscapes in a series of 23 consecutive kidney cancer patients. We discovered that a high infiltration with CD8 + T cells was not dependent on the number of driver mutations but rather on the presence of specific mutational events, namely pathogenic mutations in *PTEN* or *BAP1*. This observation encouraged us to compare mechanisms of T cell suppression in the context of four different genetic patterns, i.e., the presence of multiple drivers, a *PTEN* or *BAP*1 mutation, or the absence of detectable driver mutations. We found that ccRCCs harboring a *PTEN* or *BAP1* mutation showed the lowest level of Granzyme B positive tumor-infiltrating lymphocytes (TILs). A multiplex immunofluorescence analysis revealed a significant number of CD8 + TILs in the vicinity of CD68 + macrophages/monocytes in the context of a *BAP1* mutation but not in the context of a *PTEN* mutation. In line with this finding, direct interactions between CD8 + TILs and CD163 + M2-polarized macrophages were found in *BAP1*-mutated ccRCC but not in tumors with other mutational patterns. While an absence of driver mutations was associated with more CD8 + TILs in the vicinity of FOXP3 + Tregs and CD68 + monocytes/macrophages, the presence of multiple driver mutations was, to our surprise, not found to be strongly associated with immunosuppressive mechanisms. Our results highlight the role of genetic alterations in shaping the immunological landscape of ccRCC. We discovered a remarkable heterogeneity of mechanisms that can lead to T cell suppression, which supports the need for personalized immune oncological approaches.

## Introduction

Clear cell renal cell carcinoma (ccRCC) is among the most lethal urological malignancies once metastatic. The therapeutic landscape has changed considerably in recent years, and immune checkpoint inhibitors (ICIs) alone or in combination with tyrosine kinase inhibitors (TKIs) are now widely used to treat patients with advanced ccRCC [1, 2]. Nevertheless, a large proportion of patients continues to experience treatment failure and tumor progression with lethal disease outcome. The mechanisms of resistance to ICIs are incompletely understood [3, 4].

The focus of current immunotherapies for advanced ccRCC is on intratumoral CD8 + cytotoxic T cells. However, these T cells have been reported to be frequently dysfunctional or exhausted. In line with this notion is the finding that in ccRCC, in contrast to other tumor entities, a high number of infiltrating T cells is associated with a poor prognosis [5]. T cell dysfunction and exhaustion involve chronic neoantigen exposure and T cell receptor stimulation but also extrinsic factors such as inhibitory cell populations of the tumor immune microenvironment [6]. Recent findings highlight an important role of immunosuppressive tumor-associated macrophages (TAMs) with M2 polarization in progressive T cell exhaustion in advancing ccRCC [7]. Other inhibitory cell populations of the tumor immune microenvironment include, for example, FOXP3 + regulatory T cells (Tregs) [8]. The presence of such inhibitory cell populations has the potential to impair the clinical efficacy of ICIs, even when ICI target proteins are expressed [9, 10].

The fact that ccRCC is an immunologically vulnerable tumor entity has been attributed to the high degree of genomic intratumoral heterogeneity (ITH) [11]. It is conceivable that genomic ITH originates mostly from subclonal evolution and diversification under preservation of truncal driver events [12–14]. High-confidence driver events in ccRCC include mutations in *VHL*, *PBRM1*, *SETD2*, *BAP1*, *PTEN* and others [14]. It has been suggested that an early clonal fixation of multiple drivers leads to more rapid disease progression and poorer patient survival [14]. Whether and to what extent genetic events shape the immunological landscape of ccRCC and hence potentially affect the therapeutic efficacy of ICIs is incompletely understood.

In the present proof-of-concept study, we show that specific driver mutations rather than the overall number of driver events may influence the frequency of tumor-infiltrating lymphocytes (TILs), in particular CD8 + TILs, in ccRCC. Moreover, we discovered a remarkable inter- as well as intratumoral heterogeneity of possible T cell inhibitory mechanisms.

## Patients and methods

### Patient samples and targeted next-generation sequencing (NGS)

**Fig. 1:**
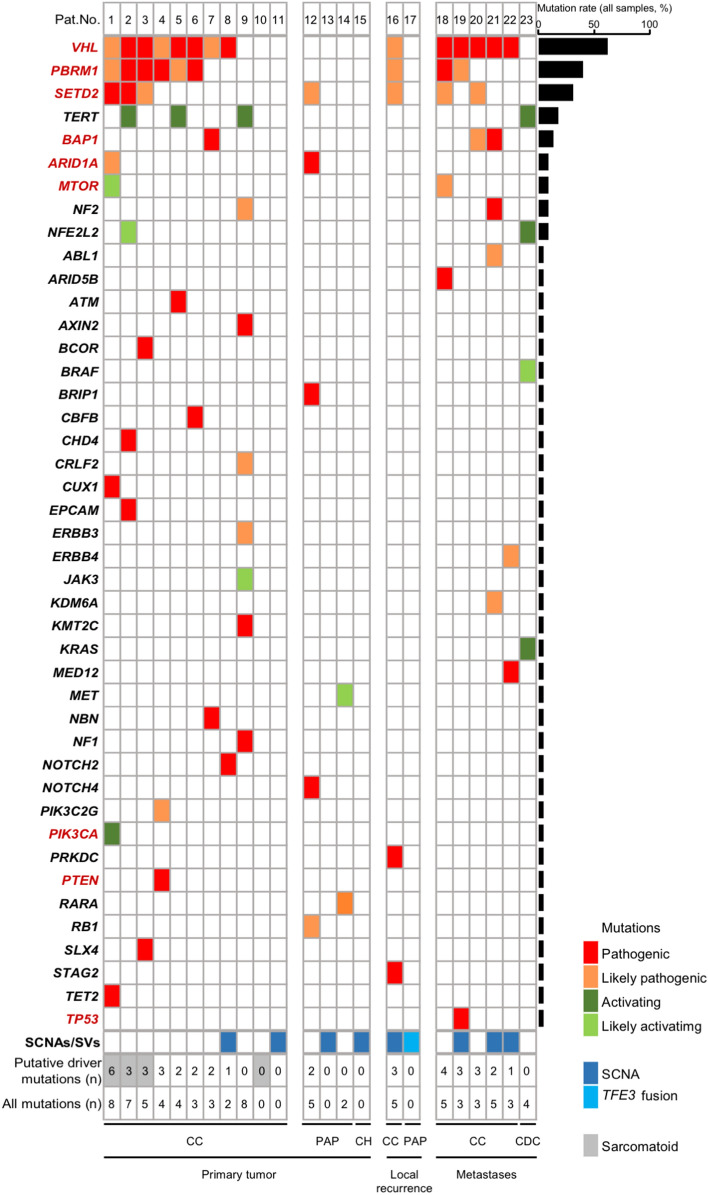
Mutational landscape of a series of 23 consecutive RCCs. Driver genes are highlighted in red. SCNA, somatic copy number alteration; SV, structural variant; CC, clear cell; PAP, papillary; CH, chromophobe; CDC, collecting duct carcinoma

In this proof-of-concept study, formalin-fixed, paraffin-embedded (FFPE) tissue sections from 23 consecutive patients with ccRCC or non-ccRCC (nccRCC) were analyzed (Table 1). Fifteen tumor samples were collected from primary tumors, two samples from local recurrences and six tissue samples from metastatic lesions. Of 23 tumors, 17 showed a clear cell histology and six were diagnosed as nccRCC including papillary RCC type I (n = 2) or II (n = 2), chromophobe RCC (n = 1) and collecting duct carcinoma (CDC, n = 1; Fig. 1).

**Table 1:** Clinico-pathological patient characteristics

Patient characteristics	(n = 23)
Sex (m/f)	7/16
Age, years (mean)	62.8
TNM stage, n (%)*	
pT1	5 (21.7)
pT2	3 (13)
pT3	12 (52.2)
pT4	1 (4.4)
pTx	2 (8.7)
p/cN0	10 (43.5)
p/cN1	4 (17.4)
p/cNx	9 (39.1)
p/cM0	8 (34.8)
p/cM1	9 (39.13)
p/cM2	1 (4.4)
p/cMx	5 (21.7)
Fuhrman Grade, n (%)	
1	1 (4.4)
2	9 (39.1)
3	6 (26.1)
4	3 (13)
unknown	4 (17.4)
Histology, n (%)	
Clear Cell	17 (73.9)
Papillary	4 (17.4)
Chromophobe	1 (4.4)
Collecting Duct	1 (4.4)
Tissue origin, n (%)	
Primary tumor	15 (65.2)
Local recurrence	2 (8.7)
Metastatic lesion	6 (26.1)

^*^for all patients (including stage at the time of diagnosis for patients with advanced disease at the time of analysis)

FFPE tissue sections were retrieved from the tissue bank of the National Center for Tumor Diseases (NCT) Heidelberg. All tissue-based experiments in this study were in accordance with the regulations of the tissue bank as well as under approval of the Ethics Committee of the University of Heidelberg School of Medicine (206/2005, 207/2005, S-864/2019).

The tumor mutation status was determined by targeted panel sequencing using the capture-based TruSight™ Oncology 500 panel (Illumina, Cambridge, UK). This gene panel includes all relevant putative driver genes for RCC and covers 523 genes (full exonic coverage). DNA was extracted using a Maxwell 16 Research System (Promega, Madison, WI, USA), followed by quantification using the QuBit 2.0 DNA High Sensitivity Kit (Thermo Fisher Scientific, Waltham, MA, USA). Library preparation was performed as previously described [15]. DNA integrity was assessed using the Genomic DNA ScreenTape Analysis on a 4150 TapeStation System (Agilent Technologies, Santa Clara, CA, USA). To fragment DNA to a length of 90–250 bp, 80 ng DNA was sheared for 50–78 s using an ME220 Focused-Ultrasonicator (Covaris, Woburn, MA, USA). Following two target capture and purification steps, enriched libraries were amplified by 15 cycles of PCR and subsequently quality controlled using the KAPA SYBR Library Quantification Kit on a StepOnePlus qPCR system (both Thermo Fisher Scientific). Libraries were sequenced on a NextSeq 500 instrument (Illumina) to a mean coverage of × 1096 using high-output cartridge and v2 chemistry [16]. All assays were performed according to the manufacturers’ protocols [17]. Targeted NGS was performed under the approval of the Ethics Committee of the University of Heidelberg School of Medicine and after written informed consent of the patient.

### Immunohistochemical staining and tissue analysis

FFPE tissue sections were deparaffinized in xylene and rehydrated in a graded ethanol series. Antigen retrieval was performed using antigen retrieval buffer (Dako, Glostrup, Denmark) heated in a steamer. Tissue sections were incubated with primary antibodies overnight at 4 °C. Primary antibodies were directed against CD4 (Abcam, Amsterdam, The Netherlands, ab133616, 1:100 dilution), CD8 (Abcam, ab17147, 1:100), CD20 (Abcam, ab78237, 1:100), CD68 (Abcam, ab213363, 1:100), FOXP3 (Abcam, ab215206, 1:50), CD163 (Leica, Newcastle upon Tyne, NCL-L-CD163, 1:100) and Granzyme B (Abcam, ab134933, 1:100). For immunodetection, tissue specimens were incubated with biotinylated secondary goat anti-mouse IgG (Life Technologies, Darmstadt, catalog # 31800,1:200) or secondary goat anti-rabbit IgG (Abcam, ab97049, 1:200) antibodies and streptavidin-peroxidase conjugate (Merck/Sigma-Aldrich, Taufkirchen, Germany, catalog # 11089153001). Staining was performed with the DAB Substrate Kit (Abcam, ab64238). Nuclei were visualized with Hematoxylin Gill I (Sigma-Aldrich, St. Louis, MO, USA) staining before dehydration and mounting.

**Fig. 2:**
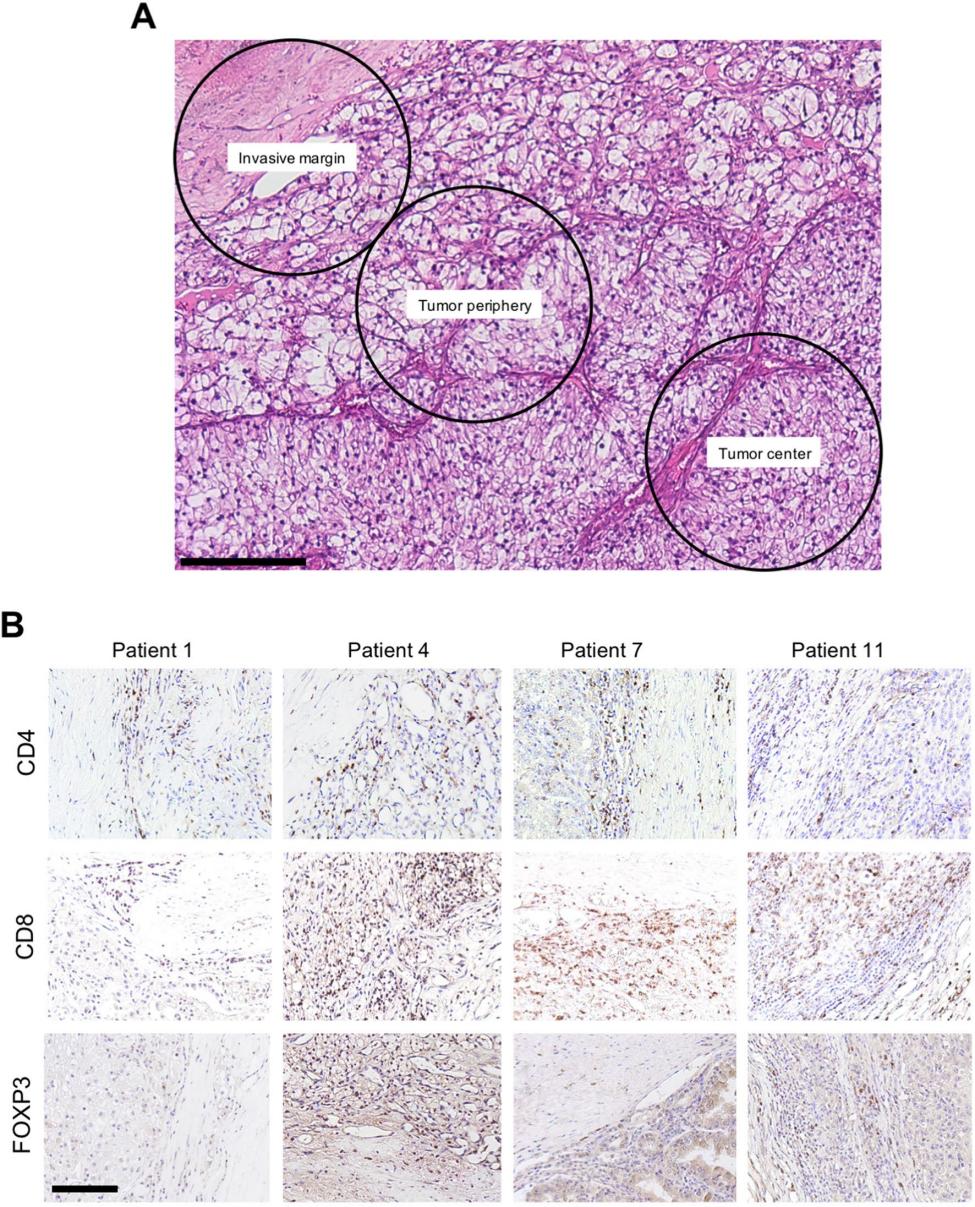
Graphic representation of tumor areas selected for semiquantitative evaluation. Circles are 500 µm in diameter and represent a 40 × HPF. Tissue stained with H&E (**A**). Representative examples of immunohistochemical staining for CD4, CD8 and FOXP3. All examples are from the invasive margin (**B**). Scale bars = 250 µm

The immunohistochemical stainings of the 23 tumors were evaluated by performing a detailed zonal semiquantitative analysis according to the International Immuno-Oncology Biomarker Working Group guidelines to TIL evaluation in solid tumors [18]. Three tumor regions were defined as follows: invasive margin (IM) with tumor border and the adjacent tumor microenvironment, tumor periphery (TP) with direct contact to the invasive margin and tumor center (TC; Fig. 2A). Positive cells in five 40 × high-power fields (HPFs) were counted for each region and each patient. In specimens from metastatic lesions, only the tumor center was analyzed. All cell counts were performed in a blinded fashion by two independent observers (J.F. and S.D.). Microphotographs were taken using a Leica DM5000 B microscope equipped with a DFC 425C digital camera (Leica, Wetzlar, Germany).

### Multispectral immunofluorescence staining and analysis

Staining procedures followed the recommendations of the Society for Immunotherapy of Cancer [19]. The basic immunophenotyping panel used in our study is well-validated and identical to the panel reported by Cillo et al. [20, 21]. Before multiplexing, single stains for each target were optimized using normal tonsil tissue since it contains all cell types relevant for our study. Normal tonsil tissue was also included as internal positive control in each staining batch.

Primary antibody concentrations, antigen retrieval conditions (pH and time) as well as fluorophore concentrations were titrated to determine optimal signal-to-noise ratio while maintaining sensitivity. More highly expressed targets were matched with lower-intensity fluorophores and vice versa. Concentration-matched isotype antibodies were used as negative controls. When designing the multiplex panel, epitopes that are degraded by successive rounds of antigen retrieval were moved to an earlier position than epitopes that become more exposed over time. It was also ensured that the staining levels of individual markers in the multiplex panel were comparable to the respective single stain.

Reproducibility across staining batches was assessed visually, and batches that were out of range were assessed separately during image analysis. Potential bleed-through issues were addressed by our phenotyping strategy and computational analysis (see also below).

FFPE tissue sections were stained using the Opal™ Polaris 7-Color Manual IHC Kit (Akoya Biosciences, Marlborough, MA, USA) according to manufacturer’s protocol. Briefly, sections were baked at 60 °C for 2 h and deparaffinized with xylene. The tissue was then rehydrated in a graded ethanol series, rinsed in deionized water and incubated in 10% neutral buffered formalin. Multispectral staining was achieved by performing consecutive rounds of antigen retrieval, primary and secondary antibody incubation as well as incubation with a tyramide signal amplification (TSA)-Opal™ dye. Antigen retrieval removes previously bound primary and secondary antibodies, while the Opal™ Polymer creates a covalent bond between the fluorophore and the tissue and thereby allows for multiple use of the same host species antibody and thus multiplex staining. For each round, microwave-based antigen retrieval was carried out using either AR6 or AR9 buffer (Akoya). Slides were blocked with blocking buffer (PerkinElmer, Waltham, MA, USA) and incubated with the respective primary antibodies (30 min at room temperature) followed by rinsing in TBS-T and incubation in HRP-conjugated, combined anti-mouse/anti-rabbit secondary antibody (PerkinElmer; 10 min at room temperature). After a further rinse in TBS-T, slides were incubated with the Opal™ fluorophore. Primary antibodies and their respective antigen retrieval and Opal™ dye were as follows: CD20 (AR9; Opal™ 520), CD8 (AR9; Opal™ 570), CD4 (AR9; Opal™ 540), FoxP3 (AR6; Opal™ 620), CD68 (AR6; Opal™ 650) and pan-CK (AR6; Opal™ 690). The staining was concluded with a final antigen retrieval (AR6) and nuclear counterstain with DAPI. Slides were mounted with antifade mounting medium (ProLong Gold, Life Technologies).

Images were acquired by the Human Immune Monitoring Shared Resource, University of Colorado, Anschutz Medical Campus. Three regions of interest (ROIs) each were selected in the IM, TP and TC using Phenochart 1.0 (PerkinElmer). High-magnification images of the ROIs were spectrally unmixed using the inForm Tissue Analysis software (Akoya) and an existing single stain library.

For image analysis, a separate algorithm was established for each marker in our panel (i.e., CD4, CD8, FOXP3, CD68). The algorithm was trained to categorize all cells in the sample as either positive or negative for the respective marker. Cells that were negative for all tested markers were categorized as “other cells.” At least 50 cells that were positive as well as 50 cells that were negative for a respective marker were included in each training set. Importantly, training was done across multiple ROIs. Visual inspection of algorithm-based phenotyping (encompassing all ROIs of a staining batch) followed by multiple rounds of retraining validated the performance of the image analysis and ensured high accuracy.

The cell phenotype categories were defined as follows: CD4 + T cells (CD4 + /CD8-/FOXP3-/CD68-), CD8 + cytotoxic T cells (CD4-/CD8 + /FOXP3-/CD68-), FOXP3 + Tregs (CD4 + /CD8-/FOXP3 + /CD68- and CD4-/CD8-/FOXP3 + / CD68-), CD68 + macrophages/monocytes (CD4-/CD8-/FOXP3-/ CD68 +).

While a subset of FOXP3 + Tregs was also CD4 + , cells in the CD4 + T cell category were only positive for CD4 and negative for all other markers. There was no further overlap between these categories.

Spatial maps were generated in R using phenoptr and phenoptrReports (both Akoya), and the number of CD8 + cells within a 20 µm radius of CD4 + , FOXP3 + or CD68 + cells was assessed.

### Statistical analysis and R packages

For statistical analyses, the non-parametric Mann–Whitney U test was performed. Differences with a *p* value ≤ 0.05 were considered statistically significant. R version 4.2.0, tidyverse version 1.3.2, ggpubr version 0.4.0, phenoptr version 0.3.2 and phenoptrReports version 0.3.3 were used.

## Results

### *PTEN* or *BAP1* mutations rather than the number of driver mutations shape the immunological landscape of RCC

Targeted NGS of the 23 ccRCCs and nccRC specimens revealed a total of 79 mutations in 43 genes, including 40 mutations in nine high-confidence ccRCC driver genes (Fig. 1) defined in analogy to the TRACERx Renal study [14]. The most commonly mutated gene was *VHL* (14 of 23 tumors, 60.9%) followed by *PBRM1* (9 of 23 tumors, 39.1%), *SETD2* (7 of 23 tumors, 30.4%), *BAP1* (3 of 23 tumors, 13%), *ARID1A* (2 of 23 tumors, 8.7%) and *MTOR* (2 of 23 tumors, 8.7%). *PIK3CA*, *PTEN and TP53* were each found to be mutated in one of the 23 tumors (4.4%). Specimens from nccRCCs either harbored some of the known ccRCC driver events, lacked mutations altogether or showed subtype-characteristic alterations such as an activating *MET* mutation in a papillary RCC (Fig. 1).

We identified a number of characteristic mutational patterns of ccRCC driver events reminiscent of the TRACERx Renal study [14] although, because no multiregion sampling was performed, without the information on clonality. Multiple drivers (i.e., two or more driver gene mutations in *PBRM1*, *SETD2*, *BAP1* or *PTEN* in addition to *VHL* [14]) were found in seven of 17 ccRCC samples (41.2%). Mutations in *VHL* and *BAP1* but in no other driver genes were found in two of 17 ccRCCs (11.8%). No driver gene mutation including in *VHL* was detected in three of 17 ccRCCs (17.6%). A mutation in *VHL* only, in the absence of other driver mutations, was detected in two of 17 ccRCCs (11.8%).

We next sought to analyze whether and to what extent patterns of driver gene mutations influence the immunological landscape. We first determined the number of TILs in a semiquantitative and spatial manner (Fig. 2A). TILs were subdivided into stroma TILs (sTILs) and intratumoral TILs (iTILs). Representative examples of immunohistochemical stainings of TIL populations are shown in Fig. 2B.

**Fig. 3:**
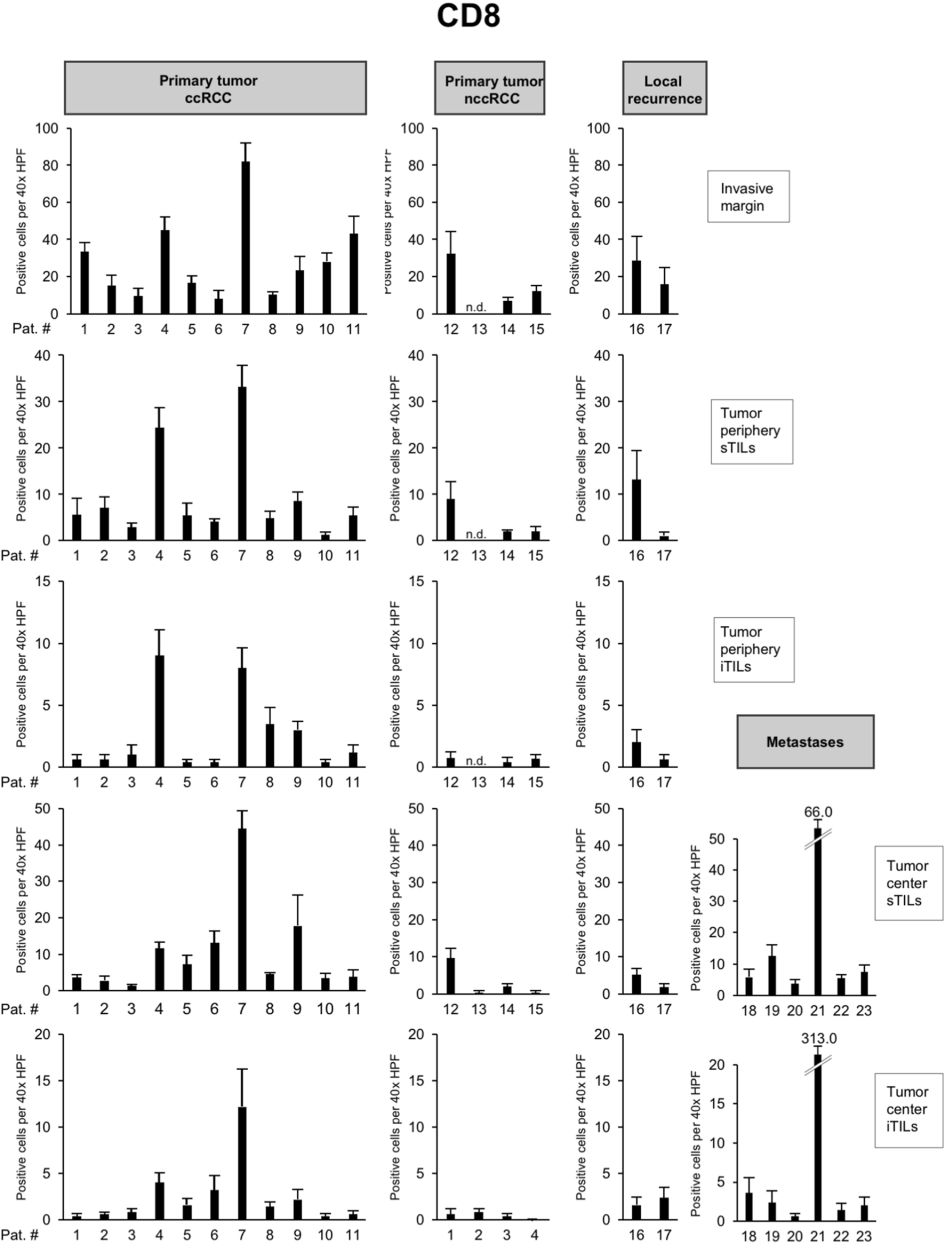
High numbers of CD8 + tumor-infiltrating lymphocytes (TILs) in ccRCCs with *BAP1* or *PTEN* mutation. Each bar represents mean and standard error of TIL counts in tumor areas indicated (see Fig. 2A), subdivided into stromal TILs (sTILs) and intratumoral TILs (iTILs) and for primary tumors, local recurrences and metastases. For each region, five 40 × HPFs were counted

The highest infiltration with CD8 + TILs in the primary tumor was consistently found in patient 7 followed by patient 4 (Fig. 3). An exception from this notion was the infiltration with iTILs in the tumor periphery, where the levels between both tumors were comparable and sTILs in the tumor center where patient 9 showed slightly higher counts than patient 4 (Fig. 3). In metastatic lesions, patient 21 showed the highest infiltration with CD8 + TILs. Both patient 7 and patient 21 harbored a pathogenic mutation in *BAP1* (p.Lys187fs*3 and p.Ser469fs*13, respectively), while patient 4 had a pathogenic mutation in *PTEN* (p.Tyr76*). Remarkably, the number of CD8 + TILs was virtually indistinguishable between patients with a high number of driver events (e.g., patient 1) or patients in which no driver mutations could be detected (e.g., patient 11; Fig. 3).

**Fig. 4:**
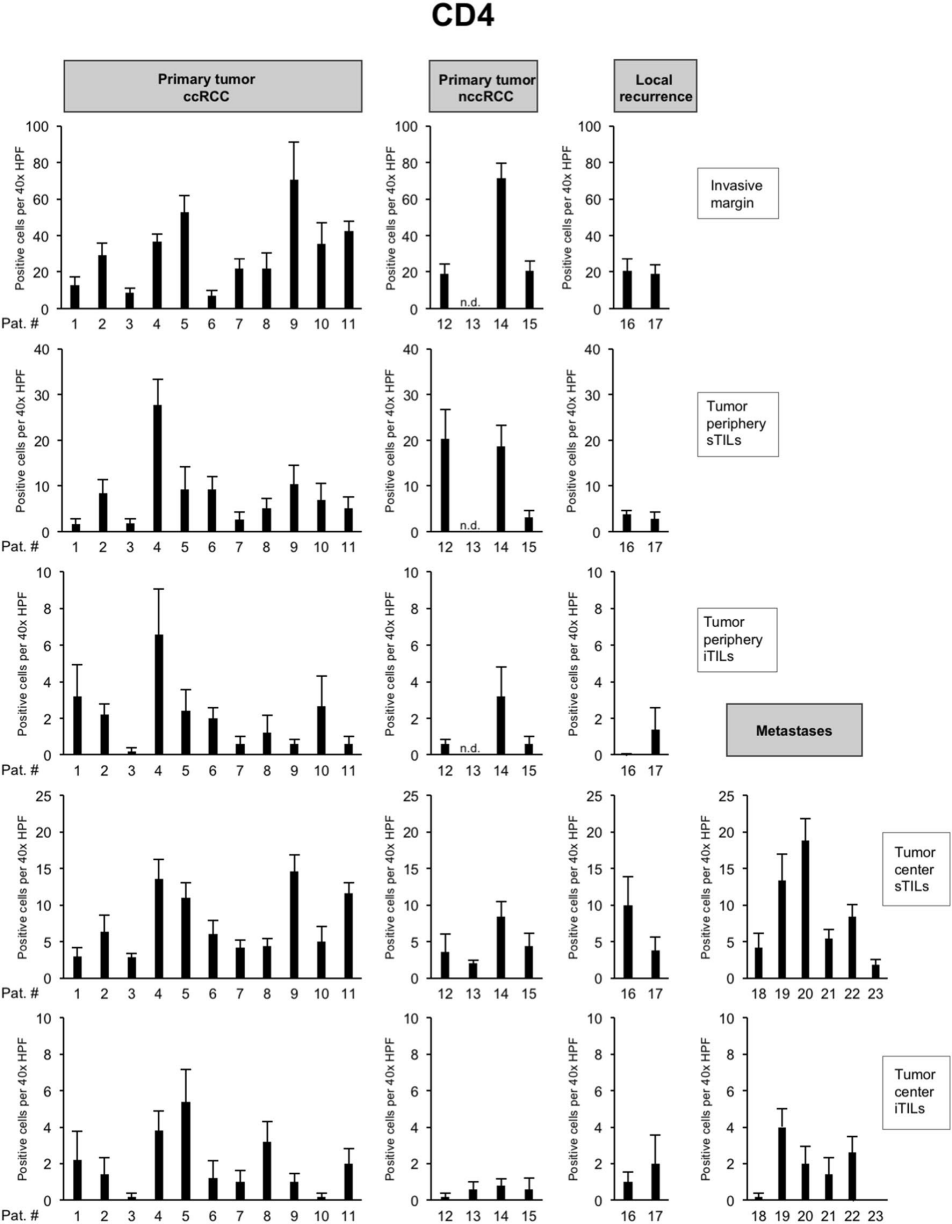
Overview of counts for CD4 + tumor-infiltrating lymphocytes (TILs). Each bar represents mean and standard error of TIL counts in tumor areas indicated (see Fig. 2A), subdivided into stromal TILs (sTILs) and intratumoral TILs (iTILs) and for primary tumors, local recurrences and metastases. For each region, five 40 × HPFs were counted

**Fig. 5:**
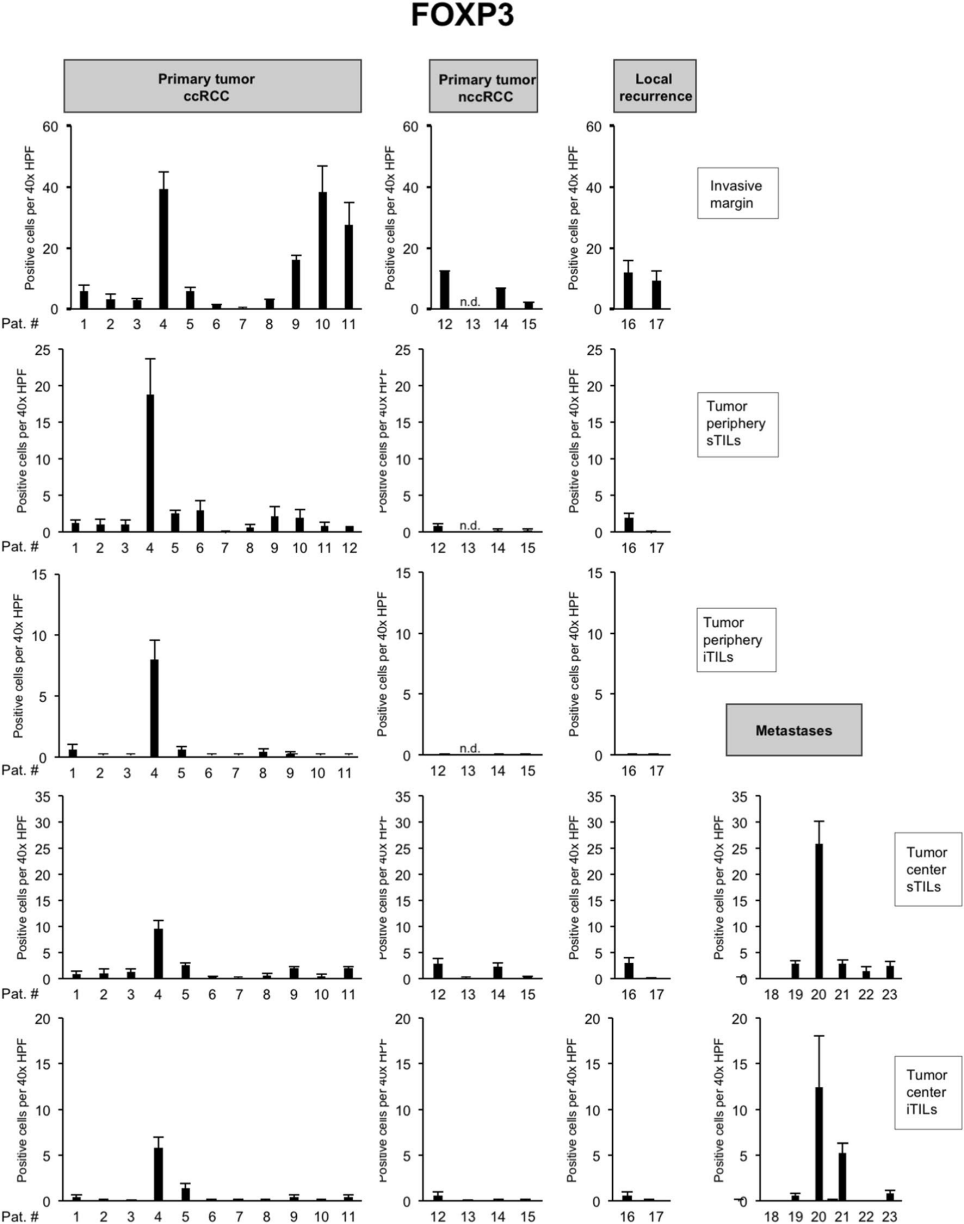
Overview of counts for FOXP3 + tumor-infiltrating lymphocytes (TILs). Each bar represents mean and standard error of TIL counts in tumor areas indicated (see Fig. 2A), subdivided into stromal TILs (sTILs) and intratumoral TILs (iTILs) and for primary tumors, local recurrences and metastases. For each region, five 40 × HPFs were counted

To characterize the immune microenvironment in the 23 tumors in greater detail, we compared the histopathological subtype as well as the mutational status to the abundance of immune cell populations including CD8 + TILs (Fig. 3) but also CD4 + TILs (Fig. 4), FOXP3 + TILs (Fig. 5), CD20 + B cells (data not shown) and CD68 + monocytes/macrophages (data not shown; Table 2).

**Table 2:** Overview of differences in the presence of immune cell populations between subgroups

Tumor region	Groups (n)	*p* values^*^				
		CD8	CD4	FOXP3	CD20	CD68
Invasive Margin	ccRCCs (12) vs. non-ccRCCs (4)	0.316	0.862	1.000	0.862	0.953
	MD or *BAP1*-mutated (6) vs. other ccRCCs (6)	0.310	0.240	0.699	0.394	0.065
	MD (5) vs. other ccRCCs (7)	0.876	0.268	0.755	0.432	0.106
	*PTEN-* or *BAP1*-mutated (2) vs. other ccRCCs (10)	0.030	0.758	1.000	0.758	0.909
	*BAP1*-mutated (1) vs. other ccRCCs (11)	0.167	1.000	0.167	0.833	0.833
Tumor periphery sTILs	ccRCCs (12) vs. non-ccRCCs (4)	0.133	0.684	0.020	0.316	0.770
	MD or *BAP1*-mutated (6) vs. other ccRCCs (6)	0.093	0.180	0.589	0.699	0.589
	MD (5) vs. other ccRCCs (7)	0.432	0.432	0.755	0.755	0.876
	*PTEN-* or *BAP1*-mutated (2) vs. other ccRCCs (10)	0.030	0.758	1.000	0.909	0.758
	*BAP1*-mutated (1) vs. other ccRCCs (11)	0.167	0.500	0.167	0.333	0.667
Tumor periphery iTILs	ccRCCs (12) vs. non-ccRCCs (4)	0.316	0.953	0.262	0.770	0.770
	MD or *BAP1*-mutated (6) vs. other ccRCCs (6)	0.310	0.937	0.937	0.310	0.818
	MD (5) vs. other ccRCCs (7)	0.755	0.876	0.755	0.202	0.755
	*PTEN-* or *BAP1*-mutated (2) vs. other ccRCCs (10)	0.030	0.606	0.606	0.485	1.000
	*BAP1*-mutated (1) vs. other ccRCCs (11)	0.333	0.667	0.667	0.667	0.500
Tumor center sTILs	ccRCCs (17) vs. non-ccRCCs (6)	0.062	0.020	0.431	0.919	0.609
	MD or* BAP1*-mutated (9) vs. other ccRCCs (8)	0.606	0.277	0.673	0.541	0.277
	MD (7) vs. other ccRCCs (10)	0.043	0.669	0.475	0.601	0.315
	*PTEN-* or *BAP1*-mutated (4) vs. other ccRCCs (13)	0.102	0.477	0.202	0.956	0.871
	*BAP1*-mutated (3) vs. other ccRCCs (14)	0.156	0.953	0.591	0.509	1.000
Tumor center iTILs	ccRCCs (17) vs. non-ccRCCs (6)	0.177	0.030	0.516	0.562	0.759
	MD or *BAP1*-mutated (9) vs. other ccRCC (8)	0.606	0.236	0.481	0.743	0.963
	MD (7) vs. other ccRCCs (10)	0.417	0.475	0.601	0.740	0.962
	*PTEN*- or *BAP1*-mutated (4) vs. other ccRCCs (13)	0.079	0.785	0.060	0.549	0.785
	*BAP1*-mutated (3) vs. other ccRCCs (14)	0.244	0.768	0.244	0.953	1.000

^*^Mann–Whitney U test. *ccRCC* clear cell renal cell carcinoma, *non-ccRCC* non-clear cell renal cell carcinoma, *MD* multiple drivers, *sTILs* stromal tumor-infiltrating lymphocytes, *iTILs* intratumoral tumor-infiltrating lymphocytes

*BAP1-* or *PTEN*-mutated ccRCCs showed significantly higher CD8 + TIL counts at the invasive margin and at the tumor periphery in comparison to other ccRCCs (*p* = 0.03; Table 2). In the presence of multiple driver mutations, higher numbers of CD8 + sTILs were detected in the tumor center, albeit only with borderline significance (*p* = 0.043). Clear cell and nccRCCs showed significant differences in the numbers of CD4 + TILs in the tumor center, both in the stroma (*p* = 0.02) and intratumorally (*p* = 0.03). Both histological subtypes also showed a significant difference in the number of FOXP3 + TILs in the stroma compartment of the tumor periphery (*p* = 0.02; Table 2). No significant differences were detected for CD20 + and CD68 + cell counts between tumors (Table 2).

Collectively, these results show a significantly higher infiltration with CD8 + TILs in *BAP1-* or *PTEN*-mutated ccRCCs both at the invasive margin and in the tumor periphery, while counts for CD4 + or FOXP3 + cells were found to depend more on the histological subtype than the mutational status.

### Inter-and intratumoral heterogeneity of T cell suppression in ccRCC

**Fig. 6:**
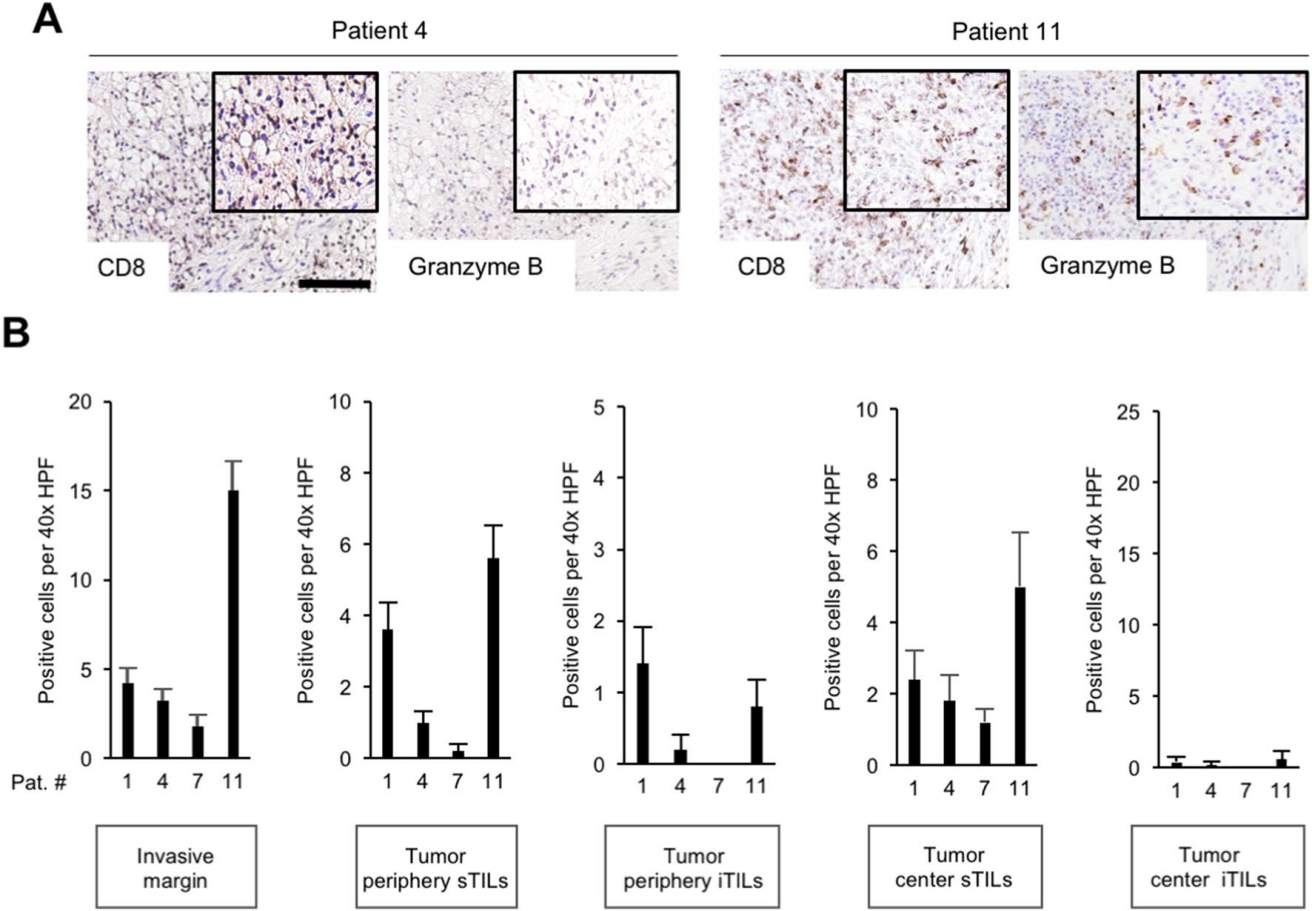
Heterogeneity of Granzyme B loss in ccRCC. Representative examples of tumors with loss of Granzyme B (patient 4) or maintained Granzyme B expression (patient 11). Scale bar = 250 μm (**A**). Semiquantitative assessment of Granzyme B + tumor-infiltrating lymphocytes (TILs) subdivided into stromal TILs (sTILs) and intratumoral TILs (iTILs) and for four patients representing different mutational patterns. Each bar represents mean and standard error of counts in the tumor areas indicated. For each region, five 40 × HPFs were counted

Next, we sought to harness our finding that specific mutations promote increased numbers of CD8 + TILs in ccRCC to explore mechanisms that have the potential to promote T cell suppression. To this end, we focused on four ccRCC patients representing different patterns of driver events (patient 1, multiple driver mutations [1^MD^]; patient 4, *PTEN*-mutated [4^*PTEN*m^], patient 7, *BAP1*-mutated [7^*BAP1*m^]; and patient 11, no driver gene mutation detected [11^ND^]). We first stained tissue specimens for Granzyme B, which is a critical component of the effector function of CD8 + cytotoxic T cells (Fig. 6). Patients 4^*PTEN*m^ and 7^*BAP1*m^ showed the lowest counts of Granzyme B positive TILs, suggesting T cell exhaustion. The number of Granzyme B positive cells was somewhat higher in patient 1^MD^, and the highest number of Granzyme B positive cells was detected in patient 11^ND^ (Fig. 6). Across tumors, the loss of Granzyme B was most pronounced in the iTIL population in the tumor center. These findings underscore the inter- as well as intratumoral heterogeneity of Granzyme B loss in ccRCC.

**Fig. 7:**
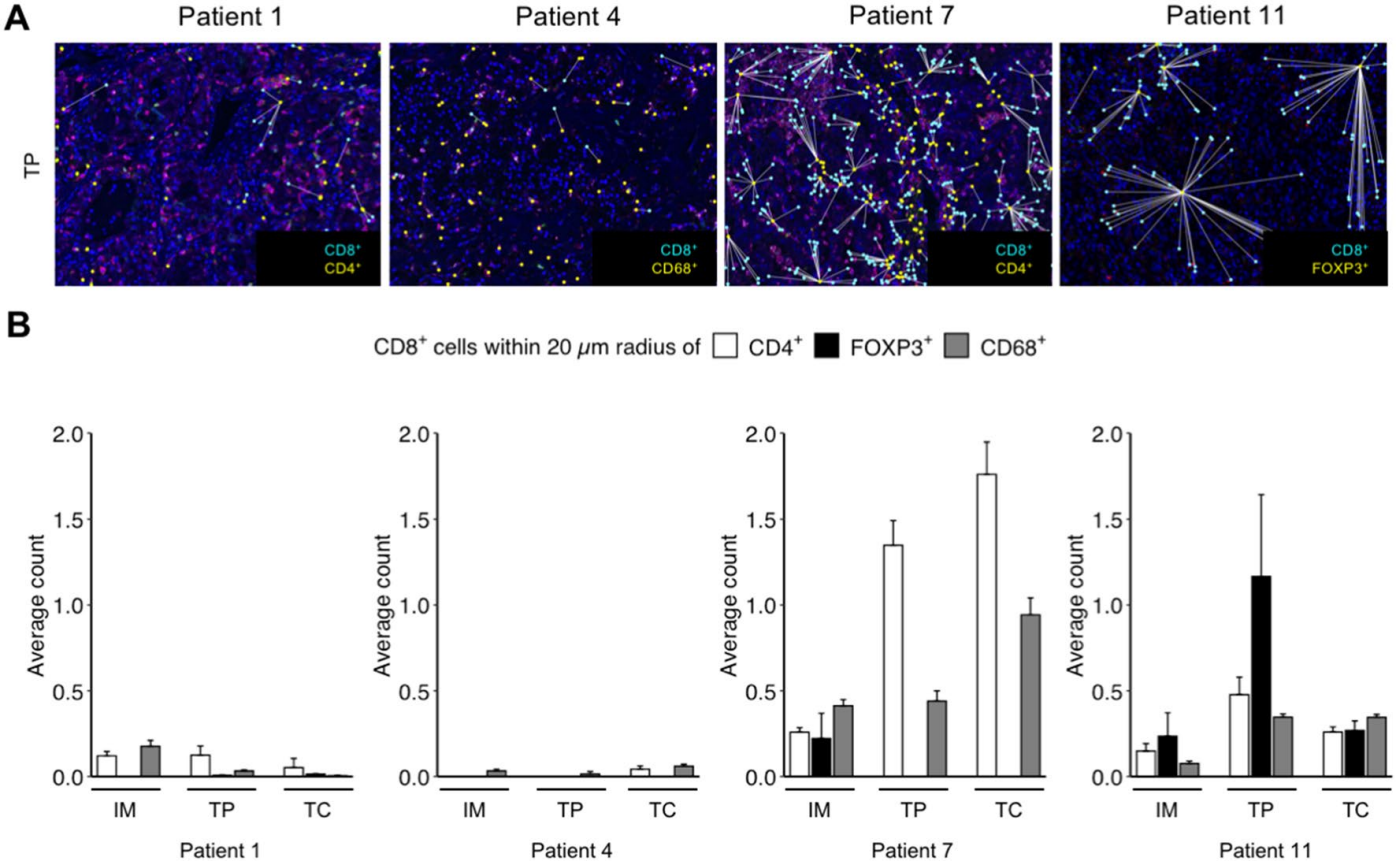
Heterogeneity of immune cell populations in the CD8 + TIL microenvironment in ccRCC. Representative microphotographs of a multiplex immunofluoresence staining for the markers indicated. White lines indicate the nearest immune cell neighbor for each CD8 + TIL. Scale bar = 250  μm (**A**). Quantification of the number of CD8 + TILs within a 20 μm radius of CD4 + , FOXP3 + or CD68 + immune cells for four ccRCC patients with different mutational patterns. Each bar represents mean and standard error of CD8 + cells obtained from three images analyzed for the different tumor regions with the exception of patient 11, where two images per region were analyzed. IM, invasive margin; TP, tumor periphery; TC, tumor center (**B**)

Next, we further analyzed possible mechanisms of CD8 + T cell suppression in the context of the tumor immune microenvironment. A multiplex immunofluorescence analysis was performed to ascertain the number of CD8 + TILs in the vicinity of CD4 + , FOXP3 + or CD68 + cells (Fig. 7A). The highest number of CD8 + TILs in the vicinity of CD4 + cells was found in patient 7^*BAP1*m^, followed by patient 11^ND^ and patient 1^MD^ (Fig. 7B). The highest counts of CD8 + TILs in the neighborhood of FOXP3 + cells were detected in patient 11^ND^ (Fig. 7B). In patient 7^*BAP1*m^, there were some CD8 + TILs in the vicinity of FOXP3 + cells, but only at the invasive margin. Patients 1^MD^ and 4^*PTEN*m^ did not show CD8 + TILs in proximity to FOXP3 + cells.

When CD8 + TILs in the vicinity of CD68 + macrophages were analyzed, the highest counts were detected in patient 7^*BAP1*m^, followed by patient 11^ND^, in all three compartments. Patient 1^MD^ showed CD8 + TILs close to CD68 + macrophages only at the invasive margin (Fig. 7B).

These results indicate a high degree of inter- as well as intratumoral heterogeneity of the CD8 + TIL immune microenvironment in ccRCC.

**Fig. 8:**
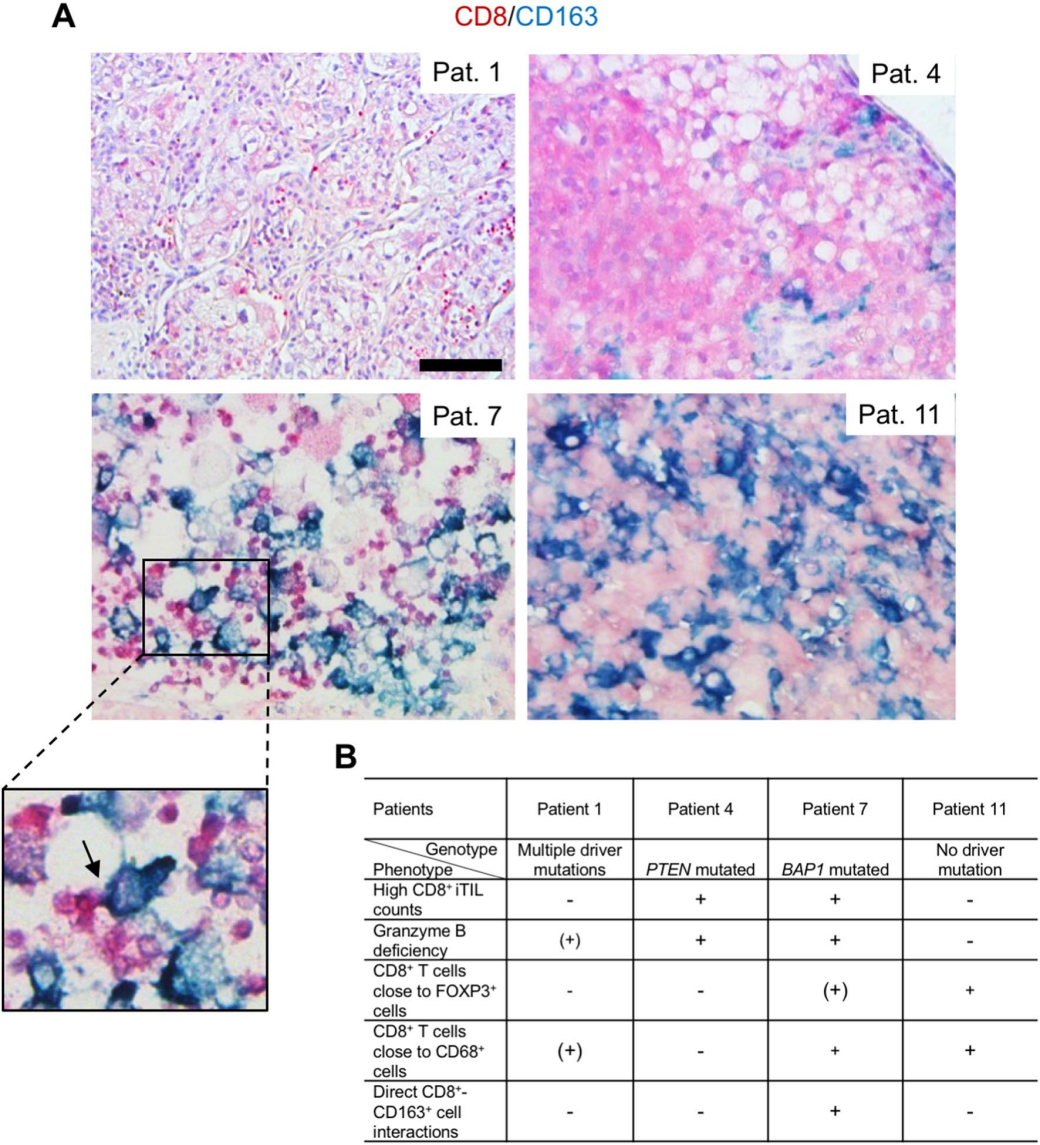
Heterogeneity of CD8 + TIL-M2 macrophage interactions in ccRCC. Double-immunohistochemical staining for CD8 (red) and CD163 (blue) of tumor specimens obtained from four ccRCC patients with different mutational patterns. Note the direct cell–cell interaction highlighted in the insert (arrow). Scale bar = 100  μm (**A**). Overview of T cell suppressive mechanisms across four ccRCC patients with different mutational patterns (**B**)

To further analyze whether CD8 + TIL-macrophage interactions involve M2 polarized immunosuppressive macrophages, we performed a double-immunohistochemical analysis for CD8 and CD163 (Fig. 8A). Direct interactions between CD8 + TILs and M2 macrophages were detected in patient 7^*BAP1*m^ and were largely absent in the other tumors.

Collectively, these findings show that different driver gene mutations are not only associated with differences in the abundance of CD8 + TILs but also with differences in potentially T cell inhibitory mechanisms. In particular, mutational inactivation of *BAP1* appears to be associated with multiple potentially T cell inhibitory mechanisms (Fig. 8B). Despite the relatively small sample size, our results highlight a remarkable inter- as well as intratumoral heterogeneity of possible mechanisms of T cell suppression.

## Discussion

Immune checkpoint inhibitors are now firmly established for the treatment of patients with advanced ccRCC. However, the majority of patients still experience disease progression and novel approaches to enhance antitumor immunity are hence urgently needed [22].

In the present proof-of-concept study, we compared the abundance of TILs to the mutational status and to mechanisms that have the potential to inhibit T cell effector functions in a series of 23 consecutive kidney cancers. T cell inhibitory mechanisms analyzed were loss of Granzyme B, the CD8 + TIL immune microenvironment and direct interactions between CD8 + TILs and immunosuppressive M2 polarized macrophages. Four genetic patterns were the focus of our study: the presence of multiple driver mutations, pathogenic mutations in *BAP1* or *PTEN*, or the absence of detectable driver mutations [14]. Remarkably, the highest infiltration with CD8 + TILs was detected in a *BAP1*-mutated ccRCC followed by a *PTEN*-mutated ccRCC, while the presence of multiple driver mutations or the absence of such mutations was virtually indistinguishable with respect to the abundance of CD8 + TILs. We found that the presence of a *BAP1* mutation was associated with all T cell inhibitory mechanisms analyzed. In contrast, other tumors showed a varying presence of these mechanisms. Despite the relatively small sample size, our results highlight the intertumoral heterogeneity of mechanisms that can suppress CD8 + T cell function. There was also intratumoral heterogeneity of these mechanisms, for example, the virtual absence of Granzyme B + iTILs in the tumor center when compared to other compartments or the presence of FOXP3 + TILs at the invasive margin but not in other tumor regions (Fig. 7B, patient 7).

Our finding of high CD8 + TIL counts in a *BAP1*-mutated tumor confirms and extends previous results. *BAP1* encodes for a deubiquitinating enzyme and is mutated in approximately 10–15% of ccRCCs [23, 24]. *BAP1*-mutated RCCs are characterized by a higher aggressiveness, increased mutational burden, poorer patient survival outcomes and are frequently "inflamed," i.e., they show an increased immune cell infiltration [23, 25]. At the same time, *BAP1*-mutated tumors have higher PD-L1 expression and have been suggested to respond better to immune checkpoint inhibition in the IMotion151 phase III clinical trial [22, 26]. Our results showing more exhausted TILs, more immunosuppressive cells in the CD8 + TIL microenvironment and direct cellular interactions between CD8 + TILs and M2 macrophages in the context of a *BAP1* mutation hence need to be reconciled with the clinical observations. One possibility is that the presence of PD-L1 overexpression may override the effects of immunosuppressive cellular interactions, which may also be more volatile. We hasten to add that this notion is based on one patient, who did not experience a tumor recurrence thus far and who has therefore not received ICIs. An association between loss of * BAP1* expression and increased infiltration with TAMs has previously been reported in uveal melanoma [27].

In contrast to *BAP1*, a mutation in *PTEN* was not found to be associated with immunosuppressive cell–cell interactions despite a significant number of CD8 + TILs. Loss of Granzyme B expression was the only mechanism affecting T cell effector function detected in this tumor. *PTEN* is mutated in approximately 5% of patients with ccRCC and its inactivation is, similar to *BAP1*, associated with genomic instability, increased mutational burden and a more unfavorable patient prognosis [28, 29]. While the influence of a *PTEN* inactivation on the number of CD8 + TILs is still under debate, there is evidence that *PTEN* loss leads to a higher abundance of M2 macrophages and Tregs in the tumor microenvironment [29]. Interestingly, a decreased expression of Granzyme B has been reported based on TCGA data similar to our finding [30]. Our patient with a *PTEN* mutation experienced disease progression upon PD-L1 blockade. Whether this outcome was related to CD8 + T cell exhaustion due to Granzyme B loss is unclear, however, it underscores that immunosuppressive mechanisms in the context of a specific mutation appear to be highly patient-individual.

One of the key findings of our study is the astonishing observation that the presence of multiple drivers or the absence of driver mutations was indistinguishable with respect to the infiltration with CD8 + TILs. In terms of immunosuppressive mechanisms, the presence of multiple drivers was not associated with any prominent feature, while the absence of driver mutations was associated with more Tregs and more CD8 + TILs in the vicinity of CD68 + macrophages, but no direct interactions with M2 polarized macrophages were detected. While the absence of a driver mutation does not formally allow the conclusion that the tumor is *VHL* wildtype, it is noteworthy that both multiple (clonal) drivers and *VHL* wildtype ccRCCs were found to have a poor prognosis in the TRACERx Renal study [14]. In our study, Patient 1 (multiple drivers) experienced rapid disease progression (< 1 year) and failure to respond to PD-1 blockade, while patient 11 (no mutation) achieved a partial remission and subsequent disease stabilization upon PD-L1 blockade in combination with a VEGFR targeting agent. We would like to add that the tumor mutational burden was comparable in our four focus patients (3.91–7.84 non-synonymous mutations per Mb) in line with a previous report showing no effect of the mutational burden on the immune cell topography [31].

The translational relevance of this study lies in highlighting the idea that targeting immunosuppressive cell populations such as M2 macrophages or Tregs may enhance the efficacy of ICIs in selected patients [32]. Given the complexity of mechanisms leading to T cell dysfunction and exhaustion, a personalized approach to immunotherapy may be required in the future to induce deeper and more durable responses in ccRCC patients.

Limitations of our study are the small number of patients, the relatively coarse characterization of the immune cell populations in comparison to other methods such as single-cell sequencing and missing information on the PD-1/PD-L1 status.

Collectively, this proof-of-concept study shows that the immune landscape in ccRCC is shaped by specific mutations, in particular in *BAP1* or *PTEN*, rather than the number of driver mutations or the mutational burden. Mechanisms affecting CD8 + T cell effector function as well as immunosuppressive cellular interactions in the tumor microenvironment appear to be highly heterogeneous and patient-specific. Our results expand the known genetic and functional inter- and intratumoral heterogeneity of ccRCC to the immune microenvironment and intrinsic as well as extrinsic mechanisms that can potentially thwart effective anticancer T cell responses.
